# dFoxO promotes Wingless signaling in *Drosophila*

**DOI:** 10.1038/srep22348

**Published:** 2016-03-03

**Authors:** Shiping Zhang, Xiaowei Guo, Changyan Chen, Yujun Chen, Jikai Li, Ying Sun, Chenxi Wu, Yang Yang, Cizhong Jiang, Wenzhe Li, Lei Xue

**Affiliations:** 1 Institute of Intervention Vessel, Shanghai 10th People’s Hospital, Shanghai Key Laboratory of Signaling and Diseases Research, School of Life Science and Technology, Tongji University, 1239 Siping Road, Shanghai 200092, China; 2 Shanghai Key Laboratory of Signaling and Diseases Research, School of Life Science and Technology, Tongji University, 1239 Siping Road, Shanghai 200092, China

## Abstract

The Wnt/β-catenin signaling is an evolutionarily conserved pathway that regulates a wide range of physiological functions, including embryogenesis, organ maintenance, cell proliferation and cell fate decision. Dysregulation of Wnt/β-catenin signaling has been implicated in various cancers, but its role in cell death has not yet been fully elucidated. Here we show that activation of Wg signaling induces cell death in *Drosophila* eyes and wings, which depends on dFoxO, a transcription factor known to be involved in cell death. In addition, dFoxO is required for ectopic and endogenous Wg signaling to regulate wing patterning. Moreover, dFoxO is necessary for activated Wg signaling-induced target genes expression. Furthermore, Arm is reciprocally required for dFoxO-induced cell death. Finally, dFoxO physically interacts with Arm both *in vitro* and *in vivo*. Thus, we have characterized a previously unknown role of dFoxO in promoting Wg signaling, and that a dFoxO-Arm complex is likely involved in their mutual functions, e.g. cell death.

The Wnt/β-catenin signaling represents one of the most intensively studied pathways, which is tightly associated with cancers, especially colorectal cancer^1^,^2^. Upon binding of Wnt ligands to the receptor Frizzled (Fz) and co-receptor LDL-receptor-related protein (LRP), the extracellular signals are transduced via Disheveled (Dsh) to the destruction complex composed of Axin (Axn), Adenomatous polyposis coli (APC) and glycogen synthase kinase 3 (GSK3), thereby preventing the proteasomal degradation of the transcriptional coactivator β-catenin. Stabilized β-catenin thus translocates into the nucleus and associates with the T cell factor (TCF)/Lymphoid-enhancing factor (LEF) family of transcription factors to activate the transcription of target genes^3^,^4^,^5^. Through this mechanism, Wnt/β-catenin signaling plays a wide range of functions, including embryogenesis, organ maintenance, cell proliferation and cell fate decisions^3^,^6^,^7^,^8^. Wnt signaling is also reported to regulate cell death during *Drosophila* retina and ommatidia development^9^,^10^,^11^, yet the underlying mechanism has not been fully elucidated, and the downstream regulators remain elusive.

The Forkhead box O (FoxO) transcription factors belong to the large forkhead family proteins, which are characterized by a winged helix DNA binding domain called ‘Forkhead box’^12^,^13^. The FoxO proteins have been conserved from *C. elegans* to mammals^14^. While there is only one *FoxO* gene in invertebrates, four members have been identified in mammals including *FoxO1*, *FoxO3a*, *FoxO4* and *FoxO6*^13^,^15^. FoxO1, 3a, and 4 are ubiquitously expressed, whereas FoxO6 expression is confined to the brain^12^. FoxO activity is negatively regulated by the Insulin/PI3K/Akt signaling pathway. Activation of Akt (also known as protein kinase B) phosphorylates FoxO and results in nuclear exclusion of FoxO, thereby inhibits its transcriptional activity^14^,^15^,^16^. However, stress conditions, such as high levels of reactive oxygen species (ROS) or deprivation of growth factors, promote the nuclear localization of FoxO and induce its target genes expression^14^. FoxO is critically involved in a variety of physiological processes including cell cycle, cell death and differentiation, DNA repair, oxidative stress response and longevity^12^,^17^,^18^,^19^. Dysregulation of FoxO has been associated with many diseases, including immune defects, malignancy, diabetes and Alzheimer’s disease (AD)^13^,^20^.

β-catenin has been reported to interact with FoxO and enhance its transcriptional activity in mammalian cells and *C. elegans*^21^. On the other hand, FoxO inhibits Wnt signaling by diverting the limited pool of β-catenin from Wnt/TCF to FoxO^22^,^23^, leading to embryogenesis and bone formation defects^24^,^25^.

Here using *Drosophila melanogaster*, which has reduced genome redundancy and many available genetic tools, we found that activation of Wingless (Wg, *Drosophila* Wnt homolog) signaling induces intensive cell death in *Drosophila* eyes and wings, which depends on dFoxO (*Drosophila* FoxO homolog). In addition, dFoxo is required for Wg signaling to activate target genes expression and execute its endogenous functions in wing patterning. Furthermore, loss of *armadillo* (arm, encoding *Drosophila* β-catenin homolog) or *pangolin* (*pan*, encoding *Drosophila* TCF homolog) could also suppress dFoxO-triggered cell death, suggesting a reciprocal effect. Finally, dFoxO physically interacts with Arm, providing a molecular mechanism for the role of dFoxO in promoting Wg signaling. Thus, contradict to the previous studies that FoxO proteins inhibit Wnt signaling, our data point to a positive role of dFoxO in modulating the canonical Wg signaling in *Drosophila*, suggesting FoxO differentially regulates Wnt signaling in a cell context-dependent manner.

## Results

### Activation of Wg signaling induces cell death in *Drosophila*

**Figure 1:**
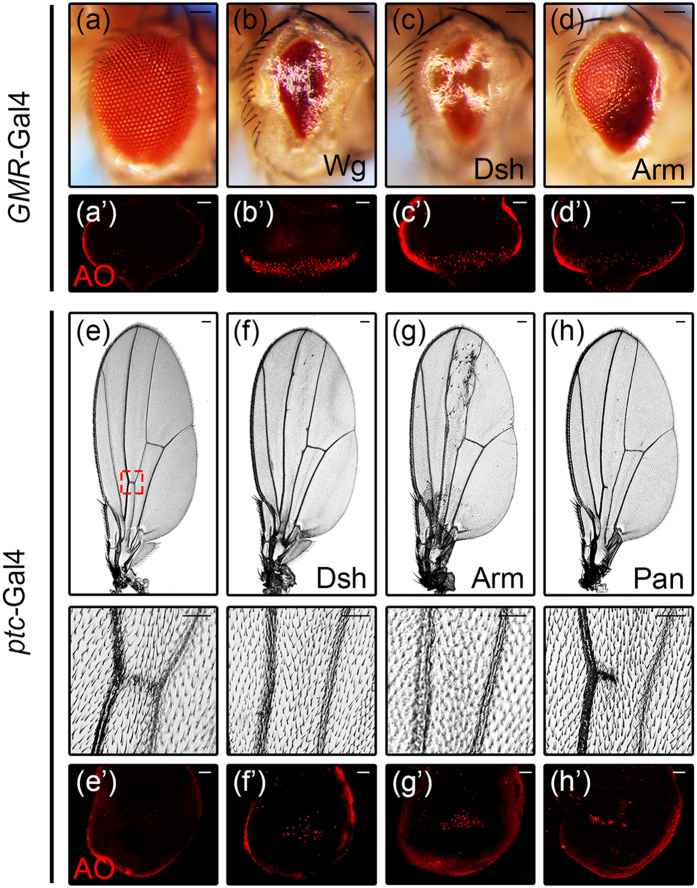
Activation of Wg signaling induces cell death in *Drosophila.* Light micrographs of *Drosophila* adult eyes and wings, and fluorescent micrographs of 3^rd^ instar eye and wing discs are shown. Compared with the *GMR*-Gal4 control **(a**,**a’**), expression of Wg, Dsh or Arm induces cell death in eye discs indicated by AO staining (**b’–d’**) and produces adult eyes with reduced size **(b–d**). Compared with the *ptc*-Gal4 control **(e**,**e’)**, expression of Dsh, Arm or Pan induces cell death in wing discs (**f’–h’**) and produces a loss-of-ACV phenotype in adult wings (**f–h**, the lower panels are high magnification of the boxed areas in upper panels). Scale bars: 100 μm in (**a**–**d**) and (**e–h**, upper panels); 50 μm in (**a’–d’**), (**e–h**, lower panels) and (**e’–h’**).

To investigate the role of Wg signaling in cell death, we expressed the representative components of this pathway in the *Drosophila* eye, and found that ectopic expression of Wg, Dsh or Arm resulted in massive cell death in 3^rd^ instar eye discs as revealed by acridine orange (AO) staining (Fig. 1a’–d’), and produced eyes with reduced size (Fig. 1a–d). In addition, ectopic expression of Dsh, Arm and Pan under *ptc*-Gal4 produced a loss of anterior cross vein (ACV) phenotype (Fig. 1e–h), accompanied by increased cell death in wing discs (Fig. 1e’–h’). Furthermore, ectopic expression of Dsh or Arm in wing discs by additional drivers including *en*-Gal4, *omb*-Gal4 and *sd*-Gal4 resulted in extensive cell death in wing pouches, as indicated by AO or TUNEL staining (Fig. S1). Collectively, these results indicate that activation of Wg signaling is able to induce cell death in *Drosophila*.

Wg signaling is necessary to provoke cell death in pupal retinas to sculpture the interommatidial lattice^9^,^10^. To investigate whether further activation of the Wg signaling could trigger more cell death in pupal retinas, we performed AO staining at 21 h after pupal formation (AFP). Consistent with previous reports^9^,^10^, extensive cell death was observed in *GMR*-Gal4 control retinas, mostly located in the anterior portion (Fig. S2a). Overexpression of Arm did not further increase cell death (Fig. S2b and S2c), suggesting the endogenous Wg signaling is optimally activated, and additional Wg signaling is not able to induce more cell death in this context.

### Loss of *dFoxO* suppresses Arm-induced cell death

**Figure 2:**
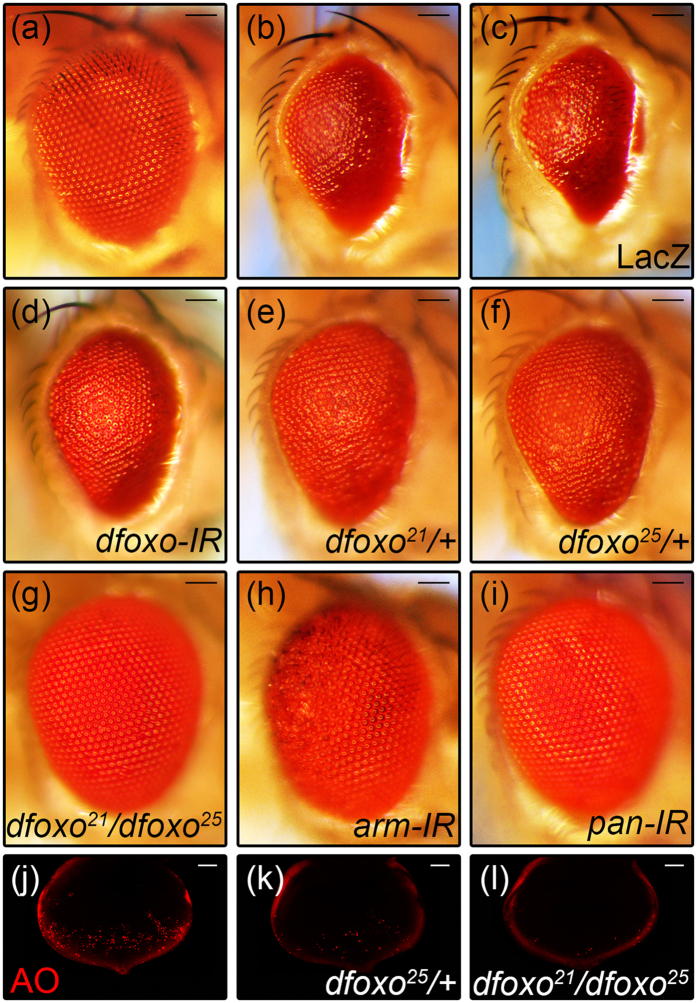
dFoxO is required for Arm-induced cell death. Light micrographs of *Drosophila* adult eyes and fluorescent micrographs of 3^rd^ instar eye discs are shown. Compared with the *GMR*-Gal4 control (**a**), expression of Arm triggers cell death and produces a small eye phenotype (**b**), which remains unaffected by expressing LacZ **(c)**, but is partially suppressed by knocking-down of *dfoxo* (**d**), or in heterozygous *dfoxo*^21^ (**e**) or *dfoxo*^25^ (**f**) background, and fully suppressed in *dfoxo*^21^*/dfoxo*^25^ mutants (**g**). As positive controls, knocking-down of *arm* (**h**) or *pan* (**i**) suppresses the *GMR* > Arm small eye phenotype. *GMR* > Arm-induced AO staining (**j**) is suppressed partially in heterozygous *dfoxo*^25^ mutants (**k**), and fully in *dfoxo*^21^*/dfoxo*^25^ trans-heterozygous mutants (**l**). Sample numbers: a, 87; b, 110; c, 100; d, 75; e, 77; f, 56; g, 75; h, 97; i, 78; j, 22; k, 14; l, 17. Scale bars: 100 μm in (**a**–**i**) and 50 μm in (**j**–**l**).

It has been reported that FoxO directly binds to β-catenin^21^, and competes with TCF for the limited pool of β-catenin, thereby inhibits Wnt signaling activity^22^,^23^. Thus we wonder whether dFoxO also inhibits Wg signaling-induced cell death in *Drosophila*. To our surprise, *GMR* > Arm-induced small eye phenotype (Fig. 2b) was suppressed partially by RNAi-mediated knocking-down of *dfoxo* (Fig. 2d), or in heterozygous *dfoxo*^21^ or *dfoxo*^25^ mutants (Fig. 2e,f), and fully in *dfoxo*^21^*/dfoxo*^25^ trans-heterozygous mutants (Fig. 2g), but remained unaffected by the expression of LacZ (Fig. 2c). As positive controls, loss of *arm* or *pan* fully suppressed *GMR* > Arm-induced small eye phenotype (Fig. 2h,i). Consistently, loss of *dfoxo* also suppressed *GMR* > Wg-induced small eye phenotype (Fig. S3). Moreover, *GMR* > Arm-induced AO staining (Fig. 2j) was suppressed partially in heterozygous *dfoxo*^25^ mutants (Fig. 2k), and fully in *dfoxo*^21^*/dfoxo*^25^ trans-heterozygous mutants (Fig. 2l). Thus, dFoxO is positively required for Wg signaling-induced cell death in *Drosophila*.

Our previous study showed Wg mediates JNK signaling induced cell death^26^, and dFoxO is reported to be a downstream transcription factor of JNK signaling^27^,^28^,^29^. To test whether JNK is reciprocally required for Wg pathway induced cell death, we overexpressed Wg, Dsh or Arm by *GMR*-Gal4 in a compromised JNK background. We found activated Wg signaling induced small eye phenotype remained unaffected by blocking JNK signaling (Fig. S4a–S4r). As a positive control, *GMR* > Egr induced small eye phenotype was effectively suppressed by abrogating JNK activity (Fig. S4s–S4x). Thus, dFoxO acts independently of the JNK pathway to mediate Wg signaling induced cell death.

### Loss of *dFoxO* suppresses Arm-induced apoptotic gene activation

**Figure 3:**
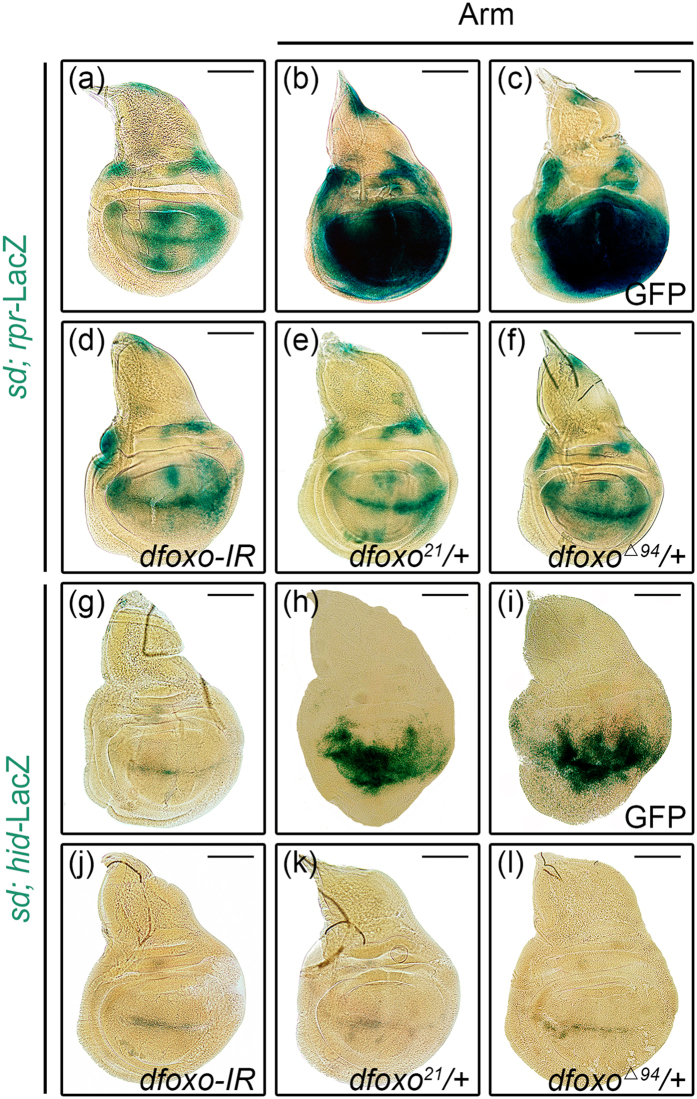
dFoxO is required for Arm-induced *hid* and *rpr* expression. Compared with the *sd*-Gal4 control (**a**,**g**), expression of Arm activates *rpr*–LacZ (**b**) and *hid*-LacZ (**h**) expression in the wing pouch, which remain unaffected by the expression of GFP (**c**,**i**), but are significantly suppressed by knocking-down of *dfoxo* (**d**,**j**), or heterozygous mutation of *dfoxo*^21^ (**e**,**k**) or *dfoxo*^Δ*94*^ **(f**,**l).** Scale bars: 100 μm.

*reaper (rpr)* and *head involution defective (hid)* are important pro-apoptotic genes regulating cell death^30^. To examine whether activated Wg signaling induces cell death through up-regulation of *rpr* and *hid*, we ectopically expressed Arm in the wing pouch driven by *sd*-Gal4. Compared with the controls (Fig. 3a,g), *rpr* and *hid* expressions were significantly up-regulated by activated Wg signaling in the wing pouch (Fig. 3b,h), which were considerably suppressed by knocking-down of *dfoxo* or in heterozygous *dfoxo* mutants (Fig. 3d–f,j–l), but remained unaffected by the expression of GFP (Fig. 3c,i). Taken together, these results suggest that dFoxO is indispensable for Wg signaling induced cell death in *Drosophila*, which contradicts to the previous reports that FoxO impedes Wnt signaling in mammalian cells^22^,^23^. Thus, it is possible that FoxO may regulate Wnt signaling differently in a tissue or cell type specific manner.

### dFoxO is required for the wing patterning functions of Wg signaling

**Figure 4:**
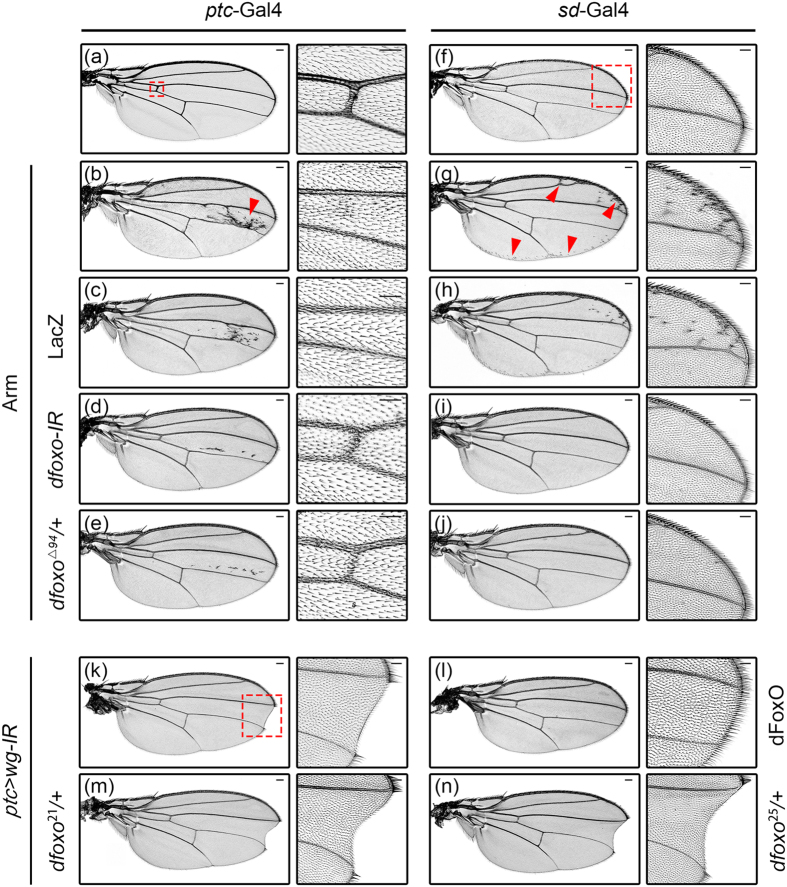
dFoxO is required for the wing patterning functions of Wg signaling. Light micrographs of *Drosophila* adult wings are shown. Compared with the *ptc*-Gal4 control (**a**, the right panel shows high magnification of the boxed area containing ACV in the left panel), expression of Arm induces ectopic bristles (indicated by the red arrow head) and loss-of-ACV phenotypes (**b**), which remain unaffected by the expression of LacZ (**c**), but are largely suppressed by RNAi-mediated knocking-down of *dfoxo* (**d**) or heterozygous mutation of *dfoxo*^Δ*94*^ (**e**). Compared with the *sd*-Gal4 control (**f**, the right panel shows high magnification of the boxed area containing anterior-distal wing margin in the left panel), expression of Arm induces ectopic bristles near the wing margin (**g**, indicated by red arrow heads), which remains unaffected by the expression of LacZ (**h**), but is suppressed by knocking-down of *dfoxo* **(i)** or heterozygous mutation of *dfoxo*^Δ*94*^ (**j**). knocking-down of *wg* by *ptc*-Gal4 generates a mild wing margin notch phenotype between the L3 and L4 veins (**k**, indicated by the red box and amplified in the right panel), which is rescued by the expression of dFoxO **(l)**, but is enhanced by heterozygous mutation of *dfoxo*^21^ **(m)** or *dfoxo*^25^ **(n)**. Sample numbers: a, 226; b, 201; c, 108; d, 197; e, 160; f,168; g, 166; h, 146; i, 130; j, 178; k,160; l, 62; m, 174; n, 226. Scale bars: 100 μm in (**a**–**n**) and 50 μm in high magnification figures.

Wg signaling is one of the profound pathways that regulate wing pattern formation. Elevation of this pathway induces ectopic bristles, whereas inhibition of which results in wing margin notches^31^,^32^. Consistent with previous reports, we found that expression of Arm driven by *ptc*-Gal4 produced ectopic bristles between L3 and L4 veins (Fig. 4b, arrow head), and generated a loss-of-ACV phenotype (Fig. 4b). Intriguingly, a similar loss-of-ACV phenotype has been reported as a result of dFoxO overexpresion (Fig. S5b)^20^. We found that both the ectopic bristles and loss-of-ACV phenotypes were considerably suppressed by loss of *dfoxo* (Fig. 4d,e), but remained unchanged by the expression of LacZ (Fig. 4c). Similarly, *sd* > Arm induced ectopic bristles near the wing margin (Fig. 4g, arrow head) were fully suppressed by loss of *dfoxo* (Fig. 4i,j), but remained unaffected by the expression of LacZ (Fig. 4h). Hence, dFoxO is required for ectopic Wg signaling-induced extra brisltes and loss-of-ACV phenotypes in the wing.

Interestingly, despite cell death in wing discs, the adult wing sizes were not significantly altered upon Arm over expression (Fig. S6a and S6b). As Wg signaling also regulates cell proliferation, we speculate that cell death might be compensated by increased cell proliferation. To address this issue, we performed PH3 staining (Fig. S6c–S6f) and BrdU incorporation (Fig. S6g–S6j) in 3^rd^ instar wing discs, but found no significant increase of cell proliferation in regions expressing Arm (Fig. S6k and S6l). Thus, other mechanism may exist to cope with ectopic Wg induced cell death and maintain tissue homeostasis in wing development. Alternatively, activation of cell death program may not necessary lead to cell loss, but instead, cell fate alteration. For instance, activation of apoptosis by *ptc* > Grim and *ptc* > *pelle-IR* produced the same loss-of-ACV phenotype^19^ as that of *ptc* > Dsh and *ptc* > Arm (Fig. 1f,g).

On the other hand, compromised Wg signaling impedes proliferation and results in loss of wing tissue, with wing margin notches as the most frequently observed phenotype^31^,^33^,^34^. Consistently, loss of *wg* between L3 and L4 veins by *ptc* > *wg-IR* generated a mild notch phenotype (Fig. 4k), which was suppressed by the expression of dFoxO (Fig. 4l), but enhanced in heterozygous *dfoxo*^21^ or *dfoxo*^25^ mutants (Fig. 4m,n). In addition, knock down *dfoxo* by two copies of *dfoxo-IR* also produced a weak notch phenotype in the wing margin (Fig. S5c). Hence, dFoxO is physiologically required for the wing patterning functions of endogenous Wg signaling.

### dFoxO is required for the activation of Wg pathway target genes

**Figure 5:**
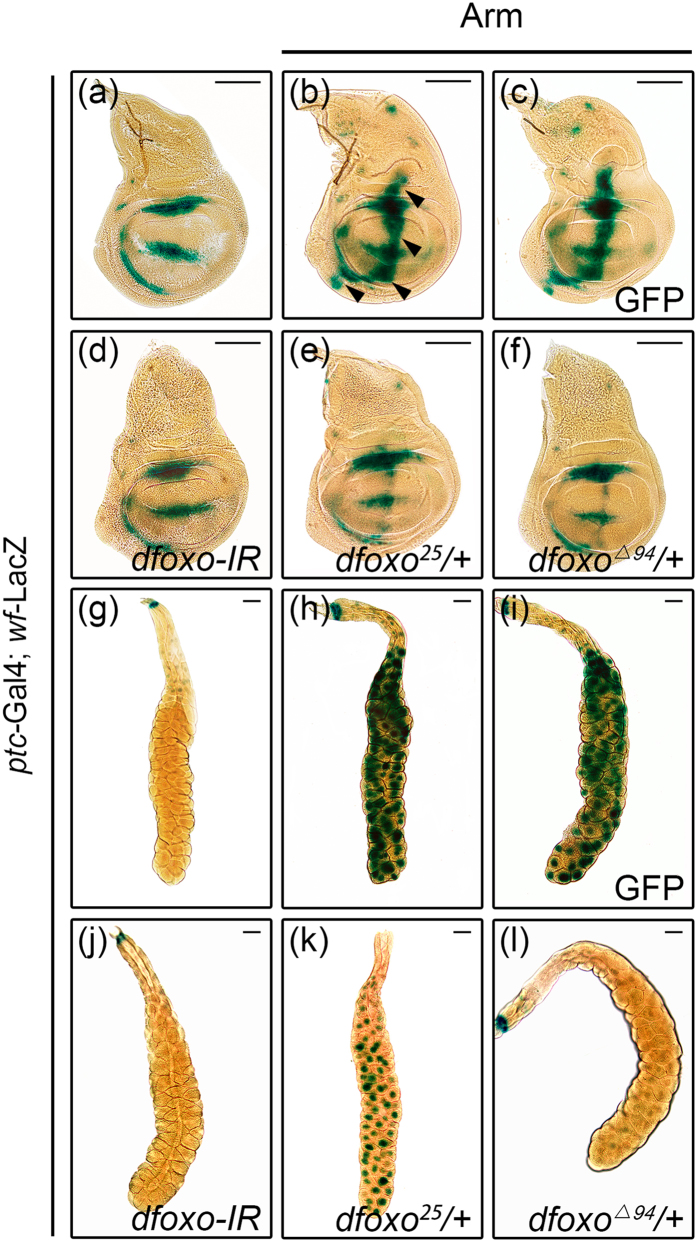
dFoxO is required for Wg target gene activation. Light micrographs of *Drosophila* 3^rd^ instar wing discs **(a–f)** and salivary glands **(g–l)** with X-Gal staining are shown. Compared with the *ptc-*Gal4 control **(a**,**g)**, ectopic Arm-induced *wf-*LacZ expression in the wing disc **(b**, indicated by black arrow heads) or salivary gland **(h)** remains unaffected by the expression of GFP **(c** and **i)**, but is significantly suppressed by knocking-down of *dfoxo* **(d**,**j)**, or heterozygous mutation of *dfoxo*^25^ **(e**,**k)** or *dfoxo*^Δ*94*^ **(f**,**l)**. Scale bars: 100 μm in (**a**–**f**) and 200 μm in (**g–l**).

To further confirm that dFoxO is required for Wg signaling activity, we checked the expression of *wingful* (*wf*), a known Wg pathway target gene that mimic *wg* expression pattern, using a *wf*-LacZ reporter (Fig. 5a)^35^. Activation of Wg signaling by *ptc* > Arm along the A/P compartment boundary of wing discs strongly up-regulated *wf* transcription (Fig. 5b). The elevated *wf* expression was significantly suppressed by the expression of a *dfoxo* RNAi (Fig. 5d) or in heterozygous *dfoxo* mutants (Fig. 5e,f), but not that of GFP (Fig. 5c). Similar results were obtained in salivary glands where loss of *dfoxo* suppressed Arm-induced *wf*-LacZ expression (Fig. 5g–l). To verify the *wf*-LacZ reporter data, we carried out qRT-PCR experiments to analyze the transcription of endogenous *wf* and another Wg signaling target gene *senseless (sens)*, which is required for bristle formation^36^. We confirmed that loss of *dfoxo* suppressed *sd* > Arm induced *wf* and *sens* expression in wing discs (Fig. S7). Thus, dFoxO is required for the transcriptional up-regulation of Wg pathway target genes.

### Arm binds to dFoxO and is reciprocally required for dFoxO-induced cell death

**Figure 6:**
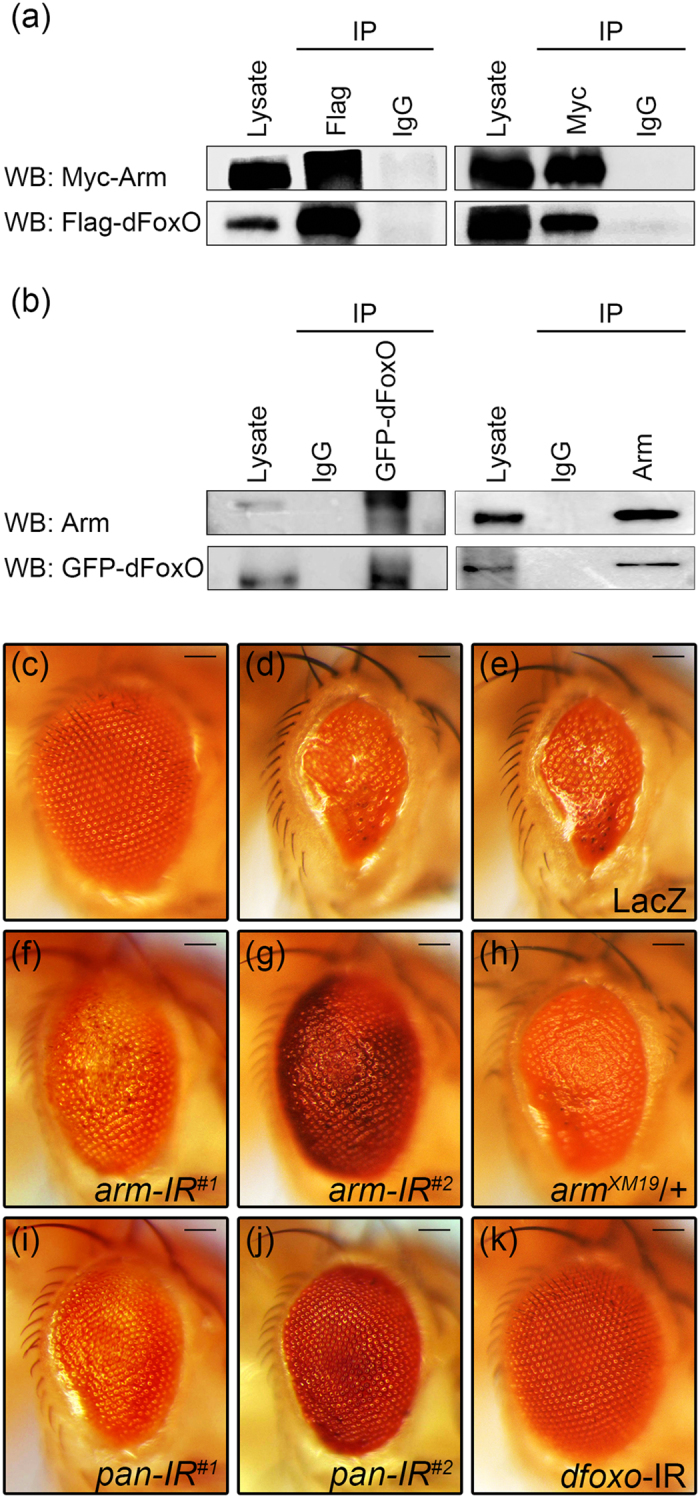
Arm binds to dFoxO and is required for dFoxO-induced cell death. **(a)** Co-immunoprecipitation between transfected Myc-Arm and Flag-dFoxO in *Drosophila* S2 cells. **(b)** Co-immunoprecipitation between ectopically expressed Arm and GFP-dFoxO in *Drosophila* eye. **(c–k)** Light micrographs of *Drosophila* adult eyes are shown. Compared with the *GMR*-Gal4 control **(c)**, expression of dFoxO triggers cell death and produces a small eye phenotype **(d)**, which remains unaffected by the expression of LacZ **(e)**, but is considerably suppressed by the expression of two independent *arm* RNAi **(f**,**g)**, or heterozygous mutation of *arm* **(h)**, or the expression of two independent *pan* RNAi **(i**,**j)**. The *GMR* > dFoxO eye phenotype is also suppressed by knocking-down of *dfoxo* **(k)**, which serves as a positive control. Sample numbers from c to k: c, 123; d, 104; e, 74; f, 67; g, 73; h, 64; i, 97; j, 106; k, 53. Scale bars: 100 μm.

When Flag-tagged dFoxO and Myc-tagged Arm were co-expressed in *Drosophila* S2 cell, Flag-dFoxO could be immunoprecipitated with Myc-Arm, and *vice versa* (Fig. 6a). Furthermore, when Arm and GFP-dFoxO were co-expressed in the fly eye, Arm could be immunoprecipitated with GFP-dFoxO, and *vice versa* (Fig. 6b), suggesting dFoxO interacts with Arm both *in vitro* and *in vivo*. Furthermore, we co-expressed Flag-dFoxO, Myc-Arm and HA-Pan in S2 cells, and performed co-IP experiments with an anti-Flag antibody. We found that the antibody not only precipitated Flag-dFoxO, but also Myc-Arm and HA-Pan (Fig. S8), suggesting all three factors exist in the same complex.

Next we examined whether Arm is reciprocally required for the function of dFoxO. Expression of dFoxO in the eye driven by *GMR*-Gal4 also induced cell death and generated a small eye phenotype^28^ (Fig. 6d). The *GMR* > dFoxO-triggered small eye phenotype was suppressed by knocking-down of *arm* or *arm* mutation (Fig. 6f–h), but remained unaffected by the expression of LacZ (Fig. 6e). Consistently, loss of *pan* also suppressed dFoxO-induced small eye phenotype (Fig. 6i,j). As a positive control, this phenotype was fully suppressed by knocking-down of *dfoxo* (Fig. 6k). Therefore, dFoxO and Arm are reciprocally required for each other’s function, possibly through their physical interaction.

## Discussion

In this work, we show that dFoxO promotes Wg signaling-induced cell death, wing pattern formation and target genes expression, while Arm is reciprocally required for dFoxO-induced cell death *in vivo*. Our data are consistent with previous study that β-catenin promotes FoxO activity^21^, but contradict to others that FoxO competes with TCF for the limited pool of β-catenin and thereby inhibits Wnt signaling activity^22^,^23^. A possible explanation for this discrepancy is that FoxO may regulate Wg/Wnt signaling in a tissue/cell type specific manner, depending on the presence of other transcriptional activating or repressing factor(s), or the different levels of FoxO and β-catenin/Arm in distinct cellular context. Importantly, while previous results were obtained mainly from the *in vitro* or gain-of-function studies, we have examined both loss-of-function and gain-of-function effects of *dfoxo* on ectopic and endogenous Wg signaling *in vivo*, and thus, the data are more likely to reflect the physiological situation. Moreover, the positive role of dFoxO in Wg signaling-induced cell death is in line with the well-known function of FoxO in promoting cell death.

The present study revealed for the first time that dFoxO acts as a vital regulator of Wg signaling-triggered cell death *in vivo*, which expands our knowledge of Wnt/Wg signaling function and the broad array of FoxOs’ regulatory activities. Both FoxO and Wnt/Wg signaling have been implicated in various cancers^2^,^3^,^12^,^13^,^37^, and a FOXO3a/β-catenin/GSK-3β signaling is essential for cell proliferation and apoptosis in prostate cancer cells^38^, further study is needed to understand their interaction in tumorigenesis.

## Materials and Methods

### *Drosophila* strains

The following lines have been described previously: *UAS-*Wg, *UAS-*Dsh, *UAS-*Arm, *UAS-*Pan, *UAS-wg-IR*, *UAS-arm-IR*^#1^, *UAS-arm-IR*^#2^, *arm*^*XM19*^, *UAS-pan-IR*^#1^, *GMR* > Egr, *UAS-*Bsk^DN^, *bsk*^*1*^, *en-*Gal4^26^; *UAS-bsk-IR*^29^; *UAS-*dFoxO-GFP-3^39^; *UAS*-*dfoxo-IR*, *dfoxo*^Δ*94*^, *ptc-*Gal4, *sd-*Gal4^20^; *UAS*-dFoxO^P^, *UAS*-dFoxO^W2^, *dfoxo*^21^, *dfoxo*^25^,^40^; *UAS-*LacZ^41^; *UAS-*GFP^42^; *GMR-*Gal4^43^; *omb-*Gal4^19^; *hid*-LacZ, *rpr*-LacZ^44^; *wf*-LacZ^35^. The *UAS*-Dcr2^D2^ (24650), *UAS-*Arm^S2^ (4783), *UAS-pan-IR*^#2^ (26743) and *bsk*^*2*^ (5283) lines were obtained from the Bloomington stock center.

### X-Gal staining

X-Gal staining was done as described^45^, discs and salivary glands were dissected in PBST at the 3^rd^ instar larva stage and fixed in 4% formaldehyde for 15 minutes, rinse the tissue once in PBST buffer containing 3.3 mM K_3_Fe(CN)_6_ and 3.3 mM K_4_Fe(CN)_6_, then incubate in the above PBST buffer with 0.2% X-gal at room temperature for 24 hours, photographs were taken under light microscope.

### AO staining

AO staining was done as described^46^, discs were dissected at the 3^rd^ instar larva stage, and stained in 1 × 10^−5^M AO solution for 5 minutes, photographs were taken under fluorescence microscope.

### TUNEL staining

TUNEL staining was done as described^26^, discs were dissected at 3^rd^ instar larva stage, and stained for TUNEL using the Fluorescein Cell Death Kit (Boster), photographs were taken under Leica confocal microscope.

### Immunostaining

Discs were dissected at 3^rd^ instar larva stage in PBS, fixed in 4% formaldehyde for 20 minutes and washed in 0.3% PBST for 3 times, then label with primary antibody overnight, and secondary antibody for 2 hours. Primary antibodies used were 1:100 mouse-anti-BrdU (Sigma) and 1:400 rabbit-anti-PH3 (Cell Signaling Technology, CST), secondary antibodies used were 1:1000 goat anti-mouse CY3 (CST) and 1:1000 goat anti- rabbit CY3 (CST). Photographs were taken under fluorescence microscope.

### BrdU incorporation

BrdU incorporation was done as described^47^, discs were dissected at 3^rd^ instar larva stage in Schneider’s medium (Sigma), incubated for 40 minutes in Schneider’s medium containing 0.2 mg/ml of BrdU, and fixed in 4% formaldehyde, then washed in PBST and hydrolyzed in 2 N HCl. After that the discs were blocked in 10% horse serum and labeled with mouse anti-BrdU and anti-mouse CY3 antibodies, photographs were taken under fluorescence microscope.

### Co-immunoprecipitation

S2 cells were transfected with indicated plasmids for 48 h, immunoprecipitation and western analyses were performed as previously described^43^, pre-cleared cell lysates were incubated with the indicated antibodies followed by precipitation with protein G Sepharose beads (Sigma). Immune complexes were washed with lysis buffer, eluted in 2 × SDS sample buffer, and then subjected to western blot using corresponding antibodies. Fly heads were cut from indicated genotypes and homogenized in lysis buffer, immunoprecipitation and western analyses were performed as above. Antibodies used in this study were as follows: mouse anti-Arm antibody (DSHB), mouse anti-Flag (Sigma), rabbit anti-GFP (CST), mouse anti-Myc (CST), rabbit anti-HA (CST) and goat anti-mouse IgG-HRP (CST).

### qRT-PCR

Wing discs were dissected at 3^rd^ instar larva, for each genotype, more than 100 discs were collected and total RNA was isolated using TRIzol (Invitrogen). qRT-PCR was performed as previously described and *rp49* was used as internal control^48^. Primer sequences were

*wf* fwd: AAGTCGAGCAATGGGCAATGAT,

rev: TGGAGGAGCGTGTCTTCTG;

*sens* fwd: CCGAAAAGGAGCATGAACTC,

rev: CGCTGTTGCTGTGGTGTACT^36^;

*rp49* fwd: CATCCGCCCAGCATACAG,

rev: CCATTTGTGCGACAGCTTAG^48^.

### Statistics

For pupal retina AO staining, wing size measurement and PH3 staining, unpaired t-text analysis was used. For qRT-PCR, one-way analysis of variance test followed by the post Dunnett test was used. A *P* value of less than 0.05 was considered significant.

## Additional Information

**How to cite this article**: Zhang, S. *et al.* dFoxO promotes Wingless signaling in *Drosophila. Sci. Rep.* **6**, 22348; doi: 10.1038/srep22348 (2016).

## Supplementary Material

Supplementary Information
