# Supplementary material for: dFoxO promotes Wingless signaling in Drosophila
Source: Sci Rep. 2016 Mar 3;6:22348. doi: 10.1038/srep22348 (PMC4776236; doi:10.1038/srep22348)
Supplement: Supplementary Information [file srep22348-s1.doc]

**dFoxO promotes Wingless signaling in *Drosophila***

Shiping Zhang^1^, Xiaowei Guo^1^, Changyan Chen^1^, Yujun Chen^1^, Jikai Li^1^, Ying Sun^1^, Chenxi Wu^1,3^, Yang Yang^1^, Cizhong Jiang^2^, Wenzhe Li^1,*^ and Lei Xue^1,*^

**Supplementary information**


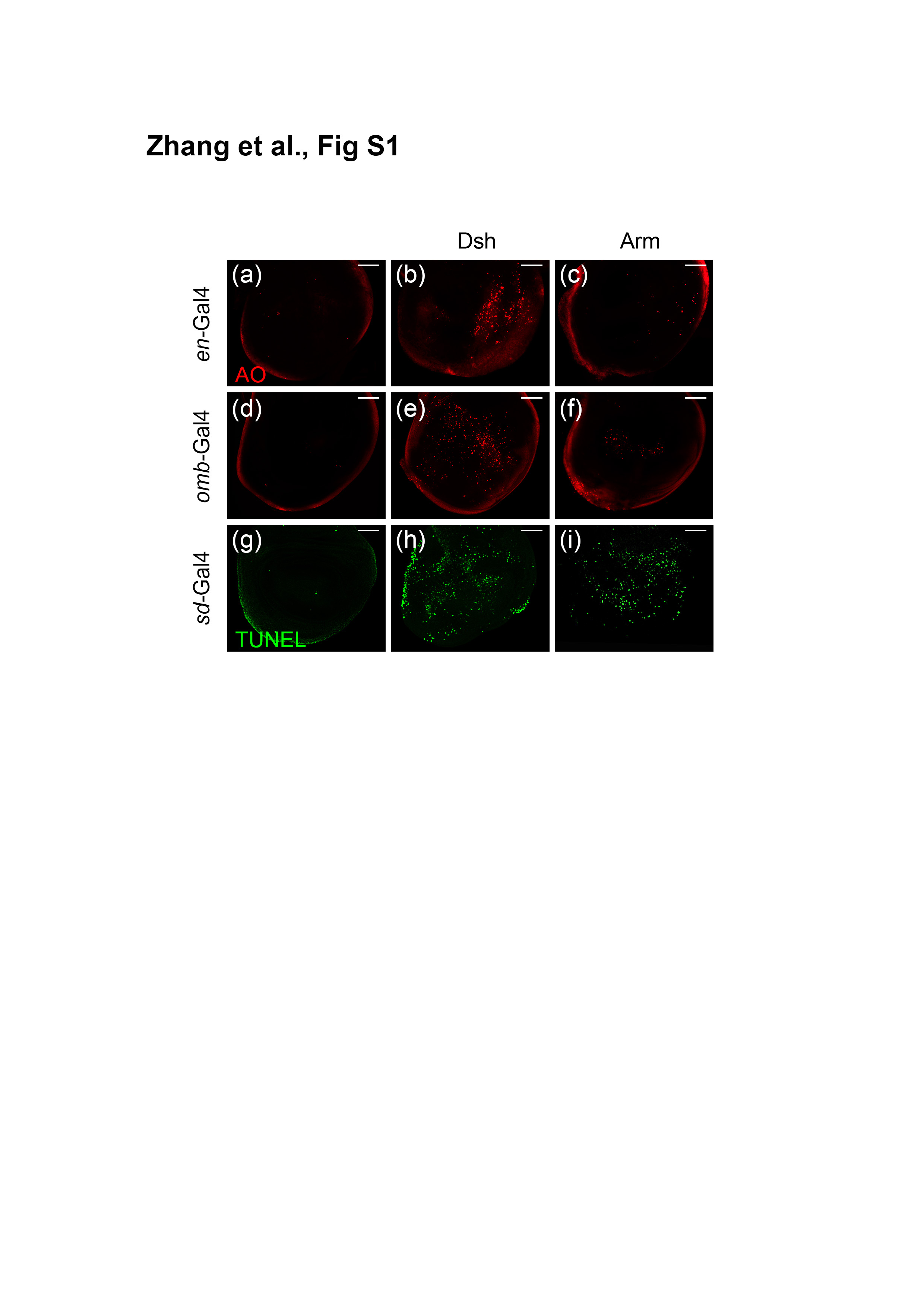
**Zhang et al., Fig S1**

**Figure S1 Activation of Wg signaling induces cell death in *Drosophila* wing discs**

Fluorescent micrographs of 3^rd^ instar wing discs are shown. Compared with the *en*-Gal4 **(a)**, *omb*-Gal4 **(d)** or *sd*-Gal4 **(g)** control, expression of Dsh **(b, e** and **h)** or Arm **(c, f** and **i)** induces extensive cell death in wing pouches, revealed by AO **(a-f)** or TUNEL **(g-i)** staining. Scale bars: 50μm.


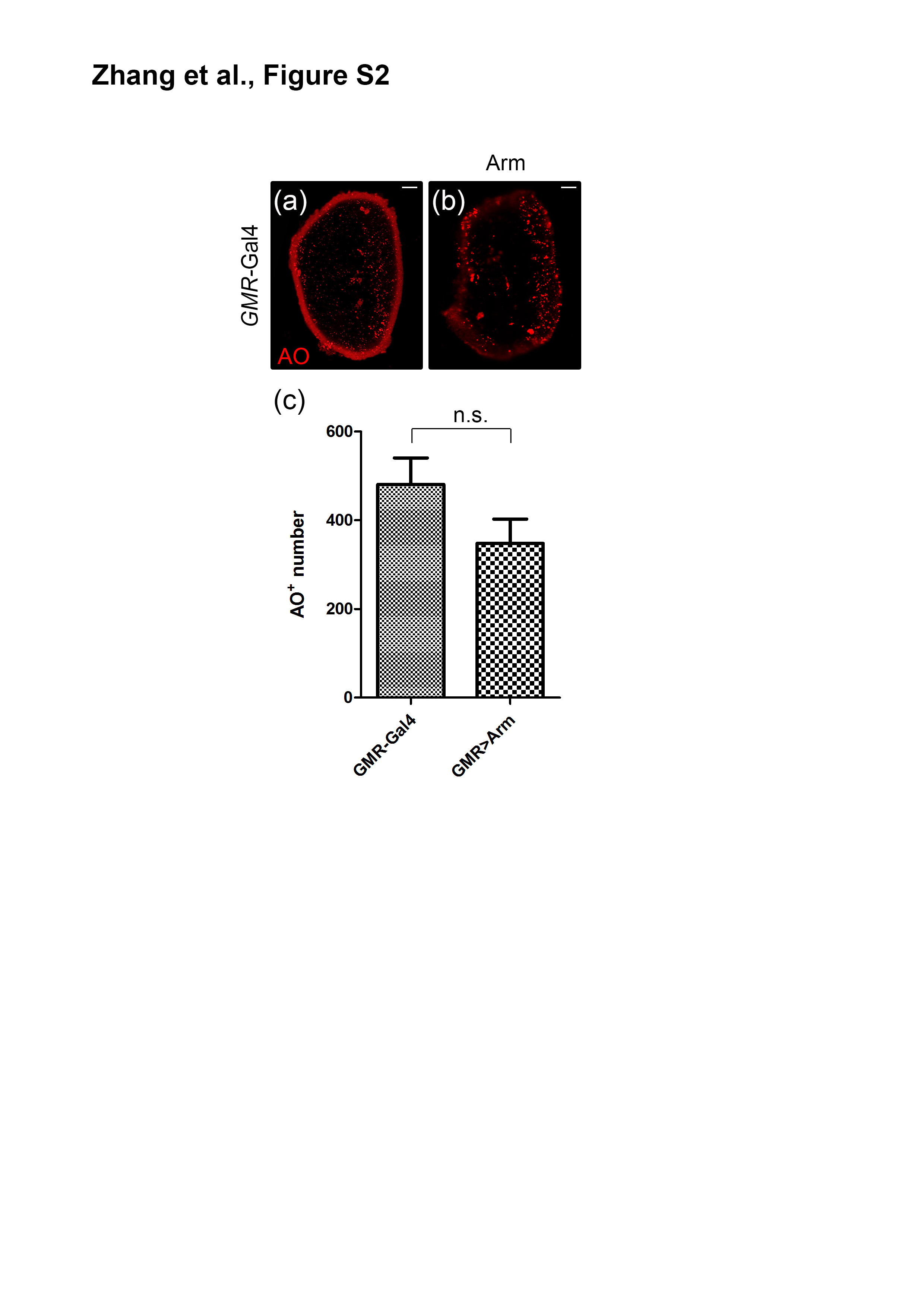
**Zhang et al., Fig S2**

**Figure S2 Activation of Arm doesn’t increase cell death in the pupal retina**

Fluorescent micrographs of 21h APF retinas with AO staining are shown. Compared with the *GMR*-Gal4 control **(a)**, overexpression of Arm doesn’t increase cell death in the pupal retina **(b)**. Statistics of AO positive cell number in figure a and b are analyzed **(c)**. Sample numbers: a, 13; b, 9. n.s., p > 0.05. Scale bars: 50μm.


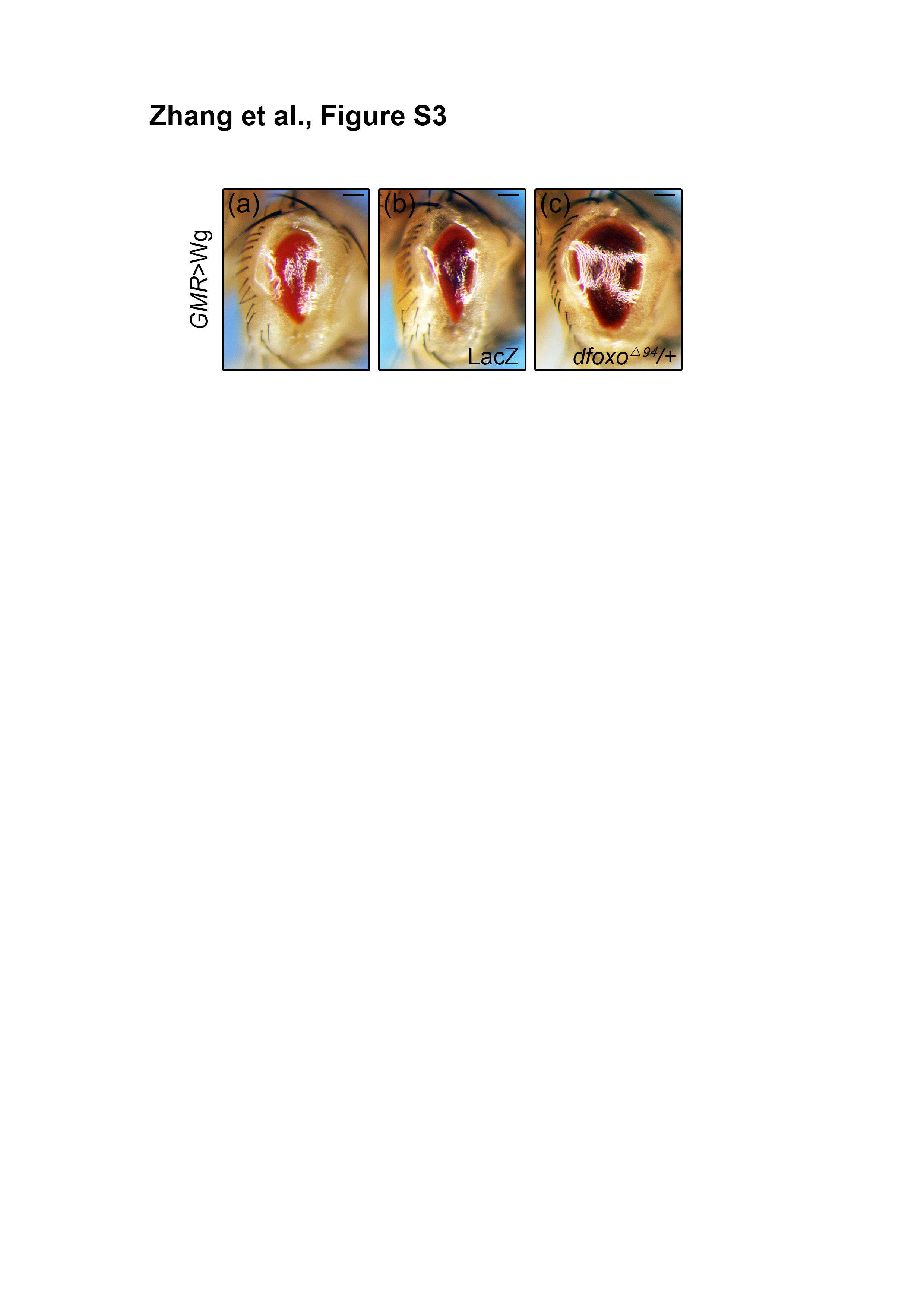
**Zhang et al., Fig S3**

**Figure S3 dFoxO is required for ectopic Wg-induced small eye phenotype**

Light micrographs of *Drosophila* adult eyes are shown. *GMR>*Wg triggered small eye phenotype **(a)** remains unaffected by the expression of LacZ **(b)**, but is suppressed by heterozygous mutation of *dfoxo^△94^* **(c)**. Sample numbers: a, 75; b, 53; c, 70. Scale bars: 100μm.


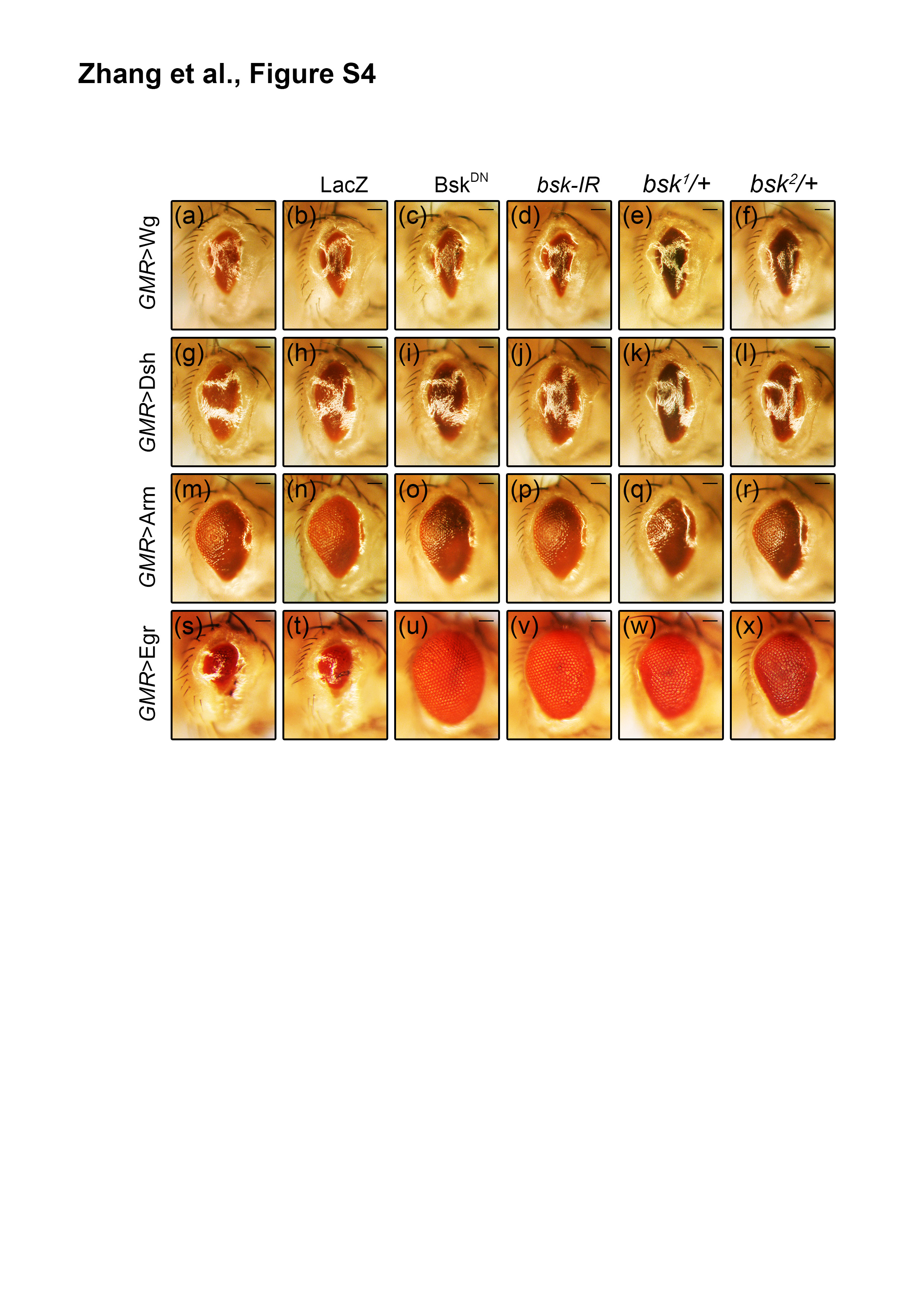
**Zhang et al., Fig S4**

**Figure S4 Wg signaling induced small eye phenotypes are JNK-independent**

Light micrographs of *Drosophila* adult eyes are shown. *GMR>*Wg **(a)**, *GMR>*Dsh **(g)** and *GMR>*Arm **(m)** induced small eye phenotypes remain unaffected by the expression of LacZ **(b, h, n)**, Bsk^DN^ **(c, i, o)**, knock down *bsk* **(d, j, p)**, heterozygous mutation of *bsk^1^* **(e, k, q)** or *bsk^2^* **(f, l, r)**. As a positive control *GMR>*Egr **(s)** induced small eye phenotype remains unaffected by the expression of LacZ **(t)**, but is strongly suppressed by the expression of Bsk^DN^ **(u)**, knock down *bsk* **(v)**, heterozygous mutation of *bsk^1^* **(w)** or *bsk^2^* **(x)**. Sample numbers: a, 95; b, 68; c, 54; d, 60; e, 52; f, 53; g, 60; h, 75; i, 51; j, 56; k, 59; l, 67; m, 69; n, 90; o, 56; p, 56; q, 51; r, 54; s, 72; t, 88; u, 67; v, 95; w, 57; x, 76. Scale bars: 100μm.


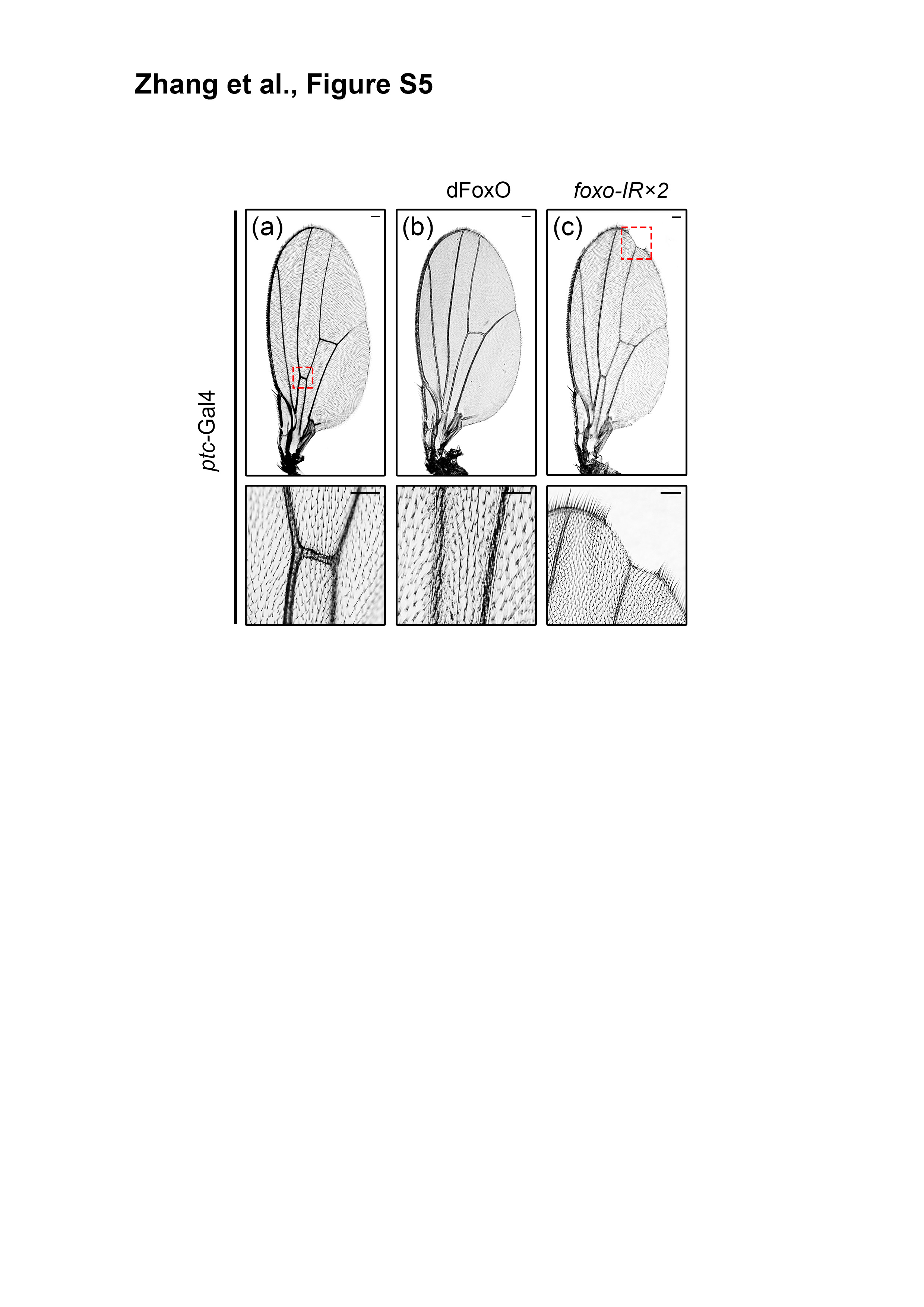
**Zhang et al., Fig S5**

**Figure S5 dFoxO mimics Wg signaling induced wing phenotypes**

Light micrographs of *Drosophila* adult wings are shown. Compared with the *ptc*-Gal4 control **(a)**, expression of dFoxO produces a loss-of-ACV phenotype **(b)**, while knock down *dfoxo* by two copies of *dfoxo-IR* generates a weak notching phenotype **(c)** in adult wings. The lower panels are high magnification of the boxed areas in upper panels**)**. Sample numbers: a, 54; b, 65; c, 6. Scale bars, 100μm in upper panels and 50μm lower panels.


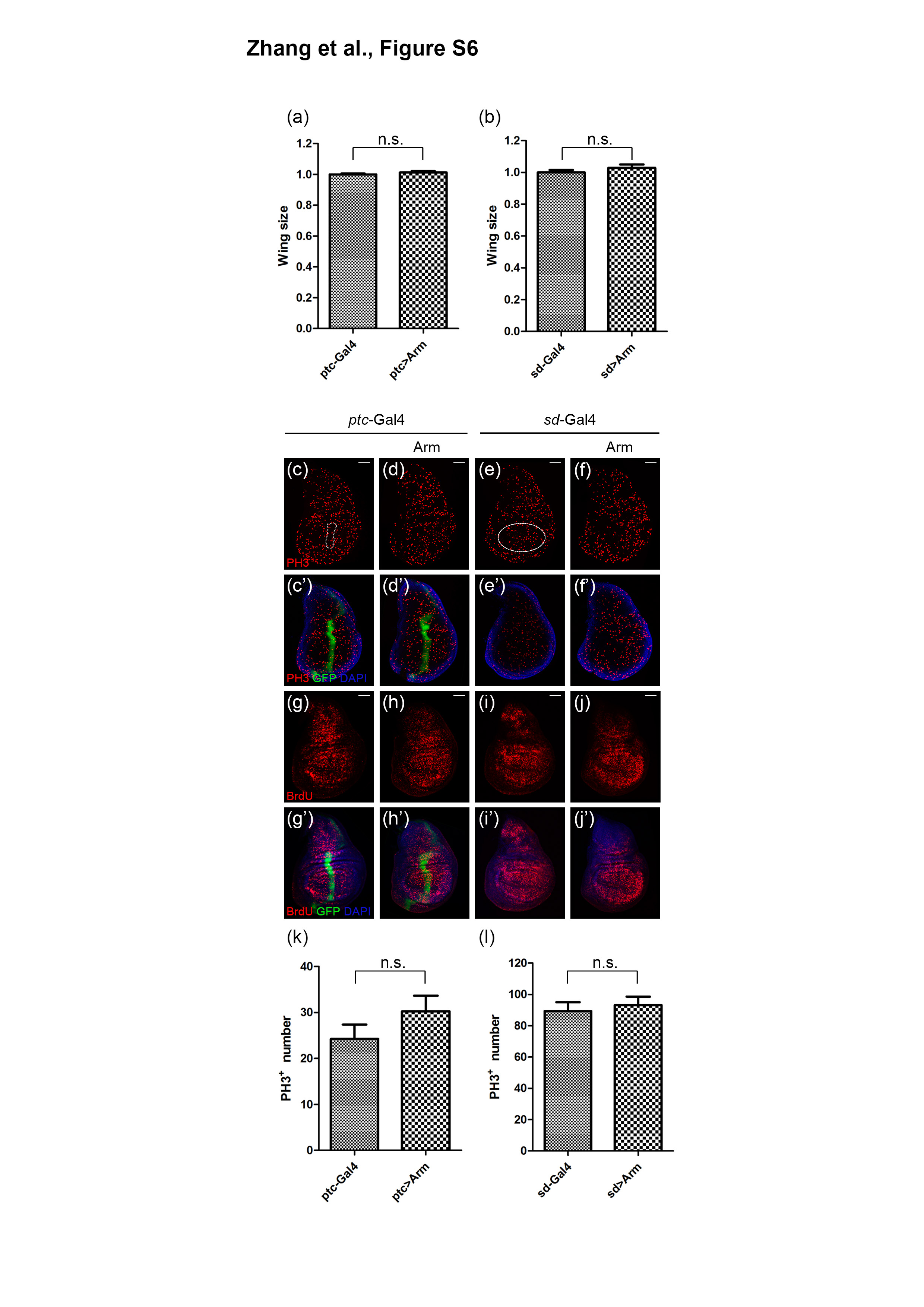
**Zhang et al., Fig S6**

**Figure S6 Activation of Arm doesn’t induce over proliferation in the wing disc**

**(a)** Statistics of *ptc-*Gal4 and *ptc>*Arm adult wing size, each value is compared to the *ptc-*Gal4 average wing size. For each genotype, more than 20 wings were analyzed. **(b)** Statistics of *sd-*Gal4 and *sd>*Arm adult wing size, each value is compared to the *sd-*Gal4 average wing size. For each genotype, more than 30 wings were analyzed. Fluorescent micrographs of 3^rd^ instar wing discs are shown from figure c to j. Compared with the *ptc*-Gal4 **(c)** or *sd*-Gal4 **(e)** control, PH3 staining shows that expression of Arm doesn’t induce over proliferation in the white dot line regions **(d** and **f)**. Compared with the *ptc*-Gal4 **(g)** or *sd*-Gal4 **(i)** control, BrdU staining shows that expression of Arm doesn’t induce over proliferation in the wing pouch **(h** and **j)**. Statistics of PH3 positive cell number in white dot line regions in figure c-f are analyzed **(k** and **l)**. Sample numbers: c, 8; d, 11; e, 15; f, 9. n.s., p > 0.05. Scale bars: 100μm.


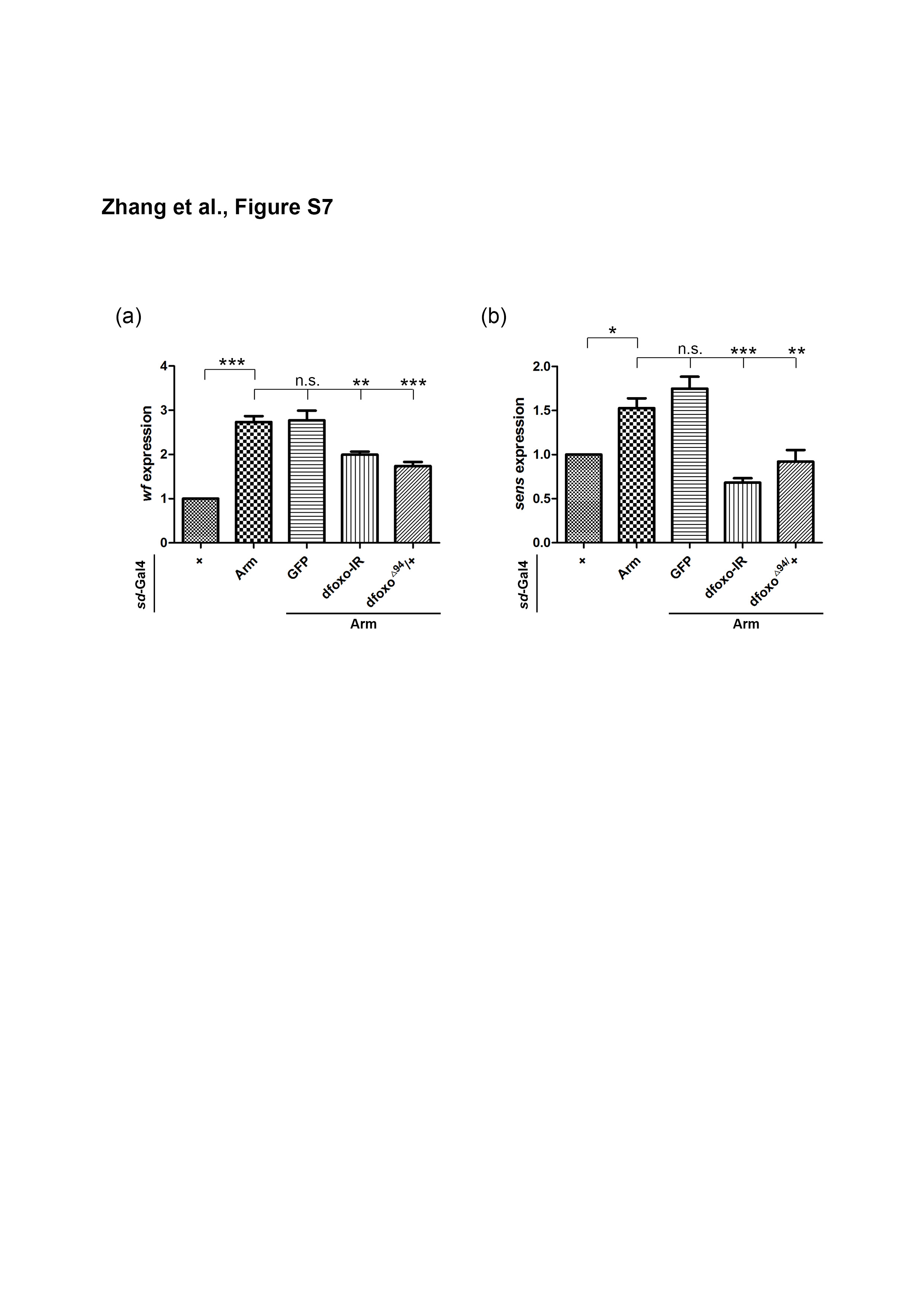
**Zhang et al., Fig S7**

**Figure S7 dFoxO is required for Wg target genes activation**

Quantification of Wg signaling target genes expression level in 3^rd^ instar wing discs by qRT-PCR. Compared with the *sd-*Gal4 control, ectopic Arm-induced *wf* **(a)** and *sens* **(b)** expression remains unaffected by the expression of GFP, but is significantly suppressed by knock down *dfoxo* or in heterozygous *dfoxo^△94^* background. Three asterisks, *p* < 0.001; two asterisks, *p* < 0.01; one asterisk, *p* < 0.05; n.s., p > 0.05.


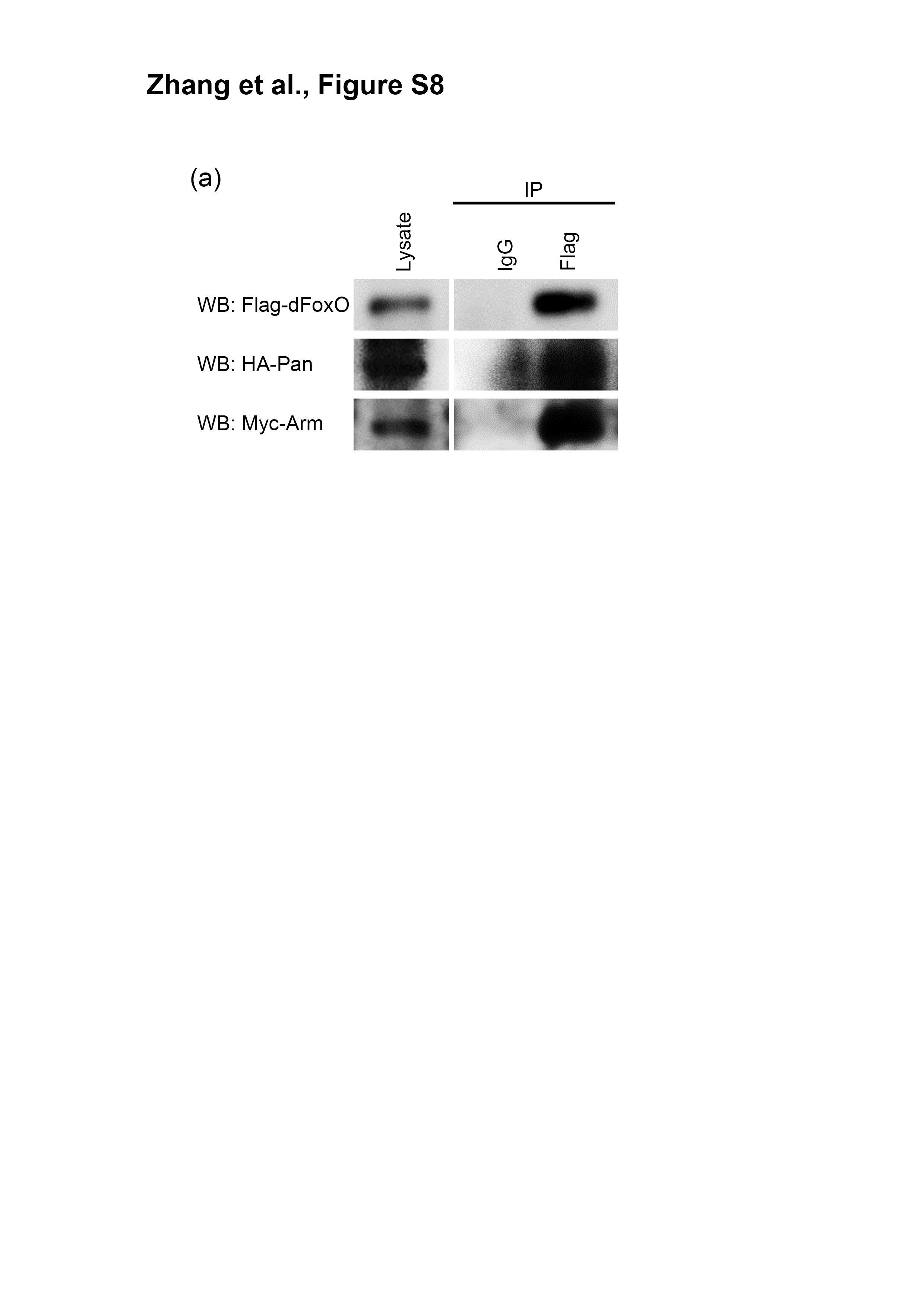
**Zhang et al., Fig S8**

**Figure S8 Arm, Pan and dFoxO form a complex**

Flag-dFoxO, Myc-Arm and HA-Pan were co-expressed in *Drosophila* S2 cells and immunoprecipitated by an anti-Flag antibody.

**Detailed Genotypes**

**Figure 1**

(a, a’) *GMR*-Gal4/+

(b, b’) *GMR*-Gal4/+; *UAS-*Wg/+

(c, c’) *GMR*-Gal4/+; *UAS-*Dsh/+

(d, d’) *GMR*-Gal4/*UAS-*Arm

(e, e’) *ptc-*Gal4/+

(f, f’) *ptc-*Gal4/+; *UAS-*Dsh/+

(g, g’) *ptc-*Gal4/*UAS-*Arm

(h, h’) *ptc-*Gal4/*UAS-*Pan

**Figure 2**

(a) *GMR*-Gal4/+

(b) *GMR*-Gal4 *UAS-*Arm/+

(c) *GMR*-Gal4 *UAS-*Arm/+; *UAS-*LacZ/+

(d) *GMR*-Gal4 *UAS-*Arm/*UAS*-Dcr2^D2^; *UAS*-*dfoxo-IR*/+

(e) *GMR*-Gal4 *UAS-*Arm/+; *dfoxo^21^*/+

(f) *GMR*-Gal4 *UAS-*Arm/+; *dfoxo^25^*/+

(g) *GMR*-Gal4 *UAS-*Arm/+; *dfoxo^21^*/*dfoxo^25^*

(h) *GMR*-Gal4 *UAS-*Arm/+; *UAS*-*arm-IR*^#1^/+

(i) *GMR*-Gal4 *UAS-*Arm/+; *UAS*-*pan-IR*^#1^/+

(j) *GMR*-Gal4 *UAS-*Arm/+

(k) *GMR*-Gal4 *UAS-*Arm/+; *dfoxo^25^*/+

(l) *GMR*-Gal4 *UAS-*Arm/+; *dfoxo^21^*/*dfoxo^25^*

**Figure 3**

(a) *sd*-Gal4/+; *rpr*-LacZ/+

(b) *sd*-Gal4/+; *UAS-*Arm/+; *rpr*-LacZ/+

(c) *sd*-Gal4/+; *UAS-*Arm/+; *rpr*-LacZ/*UAS-*GFP

(d) *sd*-Gal4/+; *UAS-*Arm/+; *rpr*-LacZ/*UAS*-*dfoxo-IR*

(e) *sd*-Gal4/+; *UAS-*Arm/+; *rpr*-LacZ/*dfoxo^21^*

(f) *sd*-Gal4/+; *UAS-*Arm/+; *rpr*-LacZ/*dfoxo^△94^*

(g) *sd*-Gal4/+; *hid*-LacZ/+

(h) *sd*-Gal4/+; *UAS-*Arm/+; *hid*-LacZ/+

(i) *sd*-Gal4/+; *UAS-*Arm/+; *hid*-LacZ/*UAS-*GFP

(j) *sd*-Gal4/+; *UAS-*Arm/+; *hid*-LacZ/*UAS*-*dfoxo-IR*

(k) *sd*-Gal4/+; *UAS-*Arm/+; *hid*-LacZ/*dfoxo^21^*

(l) *sd*-Gal4/+; *UAS-*Arm/+; *hid*-LacZ/*dfoxo^△94^*

**Figure 4**

(a) *ptc-*Gal4 *UAS-*GFP /+

(b) *ptc-*Gal4 *UAS-*GFP *UAS-*Arm/+

(c) *ptc-*Gal4 *UAS-*GFP *UAS-*Arm/+; *UAS-*LacZ/+

(d) *ptc-*Gal4 *UAS-*GFP *UAS-*Arm/*UAS*-Dcr2^D2^; *UAS*-*dfoxo-IR*/+

(e) *ptc-*Gal4 *UAS-*GFP *UAS-*Arm/+; *dfoxo^△94^*/+

(f) *sd-*Gal4/+

(g) *sd-*Gal4/+; *UAS-*Arm^S2^/+

(h) *sd-*Gal4/+; *UAS-*Arm^S2^/*UAS-*LacZ

(i) *sd-*Gal4/+; *UAS*-Dcr2^D2^/+; *UAS-*Arm^S2^/*UAS*-*dfoxo-IR*

(j) *sd-*Gal4/+; *UAS-*Arm^S2^/*dfoxo^△94^*

(k) *ptc*-Gal4/+; *UAS-wg-IR*/+

(l) *ptc*-Gal4/+; *UAS-wg-IR*/*UAS-*dFoxO^W2^

(m) *ptc*-Gal4/+; *UAS-wg-IR*/*dfoxo^21^*

(n) *ptc*-Gal4/+; *UAS-wg-IR*/*dfoxo^25^*

**Figure 5**

(a, g) *ptc-*Gal4/+; *wf*-LacZ/+

(b, h) *ptc-*Gal4 *UAS-*Arm/+; *wf*-LacZ/+

(c, i) *ptc-*Gal4 *UAS-*Arm/+; *wf*-LacZ/*UAS-*GFP

(d, j) *ptc-*Gal4 *UAS-*Arm/*UAS*-Dcr2^D2^; *wf*-LacZ/*UAS*-*dfoxo-IR*

(e, k) *ptc-*Gal4 *UAS-*Arm/+; *wf*-LacZ/*dfoxo^25^*

(f, l) *ptc-*Gal4 *UAS-*Arm/+; *wf*-LacZ/*dfoxo^△94^*

**Figure 6**

(b) *GMR*-Gal4 *UAS-*dFoxO-GFP-3/*UAS-*Arm

(c) *GMR*-Gal4/+

(d) *GMR*-Gal4 *UAS-*dFoxO^P^/+

(e) *GMR*-Gal4 *UAS-*dFoxO^P^/+; *UAS-*LacZ/+

(f) *GMR*-Gal4 *UAS-*dFoxO^P^/+; *UAS*-*arm-IR*^#1^/+

(g) *GMR*-Gal4 *UAS-*dFoxO^P^/+; *UAS*-*arm-IR*^#2^/+

(h) *arm^XM19^*/+; *GMR*-Gal4 *UAS-*dFoxO^P^/+

(i) *GMR*-Gal4 *UAS-*dFoxO^P^/+; *UAS-pan-IR*^#1^/+

(j) *GMR*-Gal4 *UAS-*dFoxO^P^/+; *UAS-pan-IR*^#2^/+

(k) *GMR*-Gal4 *UAS-*dFoxO^P^/*UAS*-Dcr2^D2^; *UAS*-*dfoxo-IR*/+

**Figure S1**

(a) *en-*Gal4/+

(b) *en-*Gal4/+; *UAS-*Dsh/+

(c) *en-*Gal4/*UAS-*Arm

(d) *omb-*Gal4/+

(e) *omb-*Gal4/+; *UAS-*Dsh/+

(f) *omb-*Gal4/+; *UAS-*Arm/+

(g) *sd-*Gal4/+

(h) *sd-*Gal4/+; *UAS-*Dsh/+

(i) *sd-*Gal4/+; *UAS-*Arm/+

**Figure S2**

(a) *GMR*-Gal4/+

(b) *GMR*-Gal4 *UAS-*Arm/+

**Figure S3**

(a) *GMR*-Gal4 *UAS-*Wg/+

(b) *GMR*-Gal4 *UAS-*Wg/*UAS-*LacZ

(c) *GMR*-Gal4 *UAS-*Wg/*dfoxo^△94^*

**Figure S4**

(a) *GMR*-Gal4 *UAS-*Wg/+

(b) *GMR*-Gal4 *UAS-*Wg/*UAS-*LacZ

(c) *GMR*-Gal4 *UAS-*Wg/*UAS-*Bsk^DN^

(d) *GMR*-Gal4 *UAS-*Wg/*UAS-bsk-IR*

(e) *bsk^1^*/+; *GMR*-Gal4 *UAS-*Wg/*+*

(f) *bsk^2^*/+; *GMR*-Gal4 *UAS-*Wg/*+*

(g) *UAS-*Dsh/+; *GMR*-Gal4/+

(h) *UAS-*Dsh/+; *GMR*-Gal4/*UAS-*LacZ

(i) *UAS-*Dsh/+; *GMR*-Gal4/*UAS-*Bsk^DN^

(j) *UAS-*Dsh/*+*; *GMR*-Gal4/*UAS-bsk-IR*

(k) *UAS-*Dsh/*bsk^1^*; *GMR*-Gal4/*+*

(l) *UAS-*Dsh/*bsk^2^*; *GMR*-Gal4/+

(m) *GMR*-Gal4 *UAS-*Arm/+

(n) *GMR*-Gal4 *UAS-*Arm/+; *UAS-*LacZ/+

(o) *GMR*-Gal4 *UAS-*Arm/+; *UAS-*Bsk^DN^/+

(p) *GMR*-Gal4 *UAS-*Arm/+; *UAS-bsk-IR*/+

(q) *GMR*-Gal4 *UAS-*Arm/*bsk^1^*

(r) *GMR*-Gal4 *UAS-*Arm/*bsk^2^*

(s) *GMR*-Gal4 *UAS-*Egr/+

(t) *GMR*-Gal4 *UAS-*Egr/+; *UAS-*LacZ/+

(u) *GMR*-Gal4 *UAS-*Egr/+; *UAS-*Bsk^DN^/+

(v) *GMR*-Gal4 *UAS-*Egr/+; *UAS-bsk-IR*/+

(w) *GMR*-Gal4 *UAS-*Egr/*bsk^1^*

(x) *GMR*-Gal4 *UAS-*Egr/*bsk^2^*

**Figure S5**

(a) *ptc-*Gal4/+

(b) *ptc-*Gal4/+; *UAS-*dFoxO^W2^/+

(c) *ptc-*Gal4/*UAS-*Dcr^D2^; *UAS-dfoxo-IR*/*UAS-dfoxo-IR*

**Figure S6**

(c, g) *ptc-*Gal4 *UAS-*GFP/+

(d, h) *ptc-*Gal4 *UAS-*GFP *UAS-*Arm/+

(e, i) *sd-*Gal4/+

(f, j) *sd-*Gal4/+; *UAS-*Arm^S2^/+
